# Physiological Replication of the Human Glomerulus Using a Triple Culture Microphysiological System

**DOI:** 10.1002/advs.202303131

**Published:** 2023-10-22

**Authors:** Ramin Pajoumshariati, Lorna Ewart, Ville Kujala, Raymond Luc, Samantha Peel, Adam Corrigan, Heather Weber, Bramasta Nugraha, Pernille B. L. Hansen, Julie Williams

**Affiliations:** ^1^ Bioscience Renal Research and Early Development Cardiovascular Renal and Metabolism (CVRM) BioPharmaceuticals R&D AstraZeneca Gothenburg 431 83 Sweden; ^2^ Emulate Inc. Boston MA 02210 USA; ^3^ Functional Genomics, Research and Early Development Discovery Sciences BioPharmaceuticals R&D AstraZeneca Cambridge CB21 6GH UK; ^4^ Vertex Pharmaceuticals Boston MA 02210 USA

**Keywords:** cross‐talk study, glomerulus‐on‐a‐chip, mesangium, tri‐culture model

## Abstract

The function of the glomerulus depends on the complex cell–cell/matrix interactions and replication of this in vitro would aid biological understanding in both health and disease. Previous models do not fully reflect all cell types and interactions present as they overlook mesangial cells within their 3D matrix. Herein, the development of a microphysiological system that contains all resident renal cell types in an anatomically relevant manner is presented. A detailed transcriptomic analysis of the contributing biology of each cell type, as well as functionally appropriate albumin retention in the system, is demonstrated. The important role of mesangial cells is shown in promoting the health and maturity of the other cell types. Additionally, a comparison of the incremental advances that each individual cell type brings to the phenotype of the others demonstrates that glomerular cells in simple 2D culture exhibit a state more reflective of the dysfunction observed in human disease than previously recognized. This in vitro model will expand the capability to investigate glomerular biology in a more translatable manner by the inclusion of the important mesangial cell compartment.

## Graphical abstract

A novel glomerular microphysiological system is presented incorporating all relevant cell types, including mesangial cells. Transcriptomic analysis demonstrates improved maturity and health of all cells, and a move away from the disease‐like phenotype of 2D cultures. Functional analysis shows improved barrier functional and formation of junctional contacts. This comprehensive model enhances the understanding of glomerular biology in a translational manner.

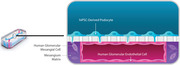

## 1 Introduction

The kidney is a major site for excretion which is achieved via selective filtration of the blood. The filtration barrier is composed of non‐diaphragmed fenestrated endothelial cells separated via a specialized basement membrane from terminally differentiated podocytes, which have interdigitating foot processes that form a zipper‐like structure termed the slit diaphragm.^[^ ^1^ ^]^ This semi‐permeable structure is structurally supported by smooth muscle‐like mesangial cells and together is defined as the renal glomerulus.^[^ ^2^ ^]^ The glomerulus plays a major role in whole body homeostasis. Due to its complex structure, it is highly susceptible to damage, such as by nephrotoxins or in disease, and is not amenable to repair. Disturbances to the glomerulus lead to the presence of proteins in the urine, which are used to diagnose glomerular dysfunction. Drug elimination may occur through the kidney and therefore can be directly influenced by glomerular function.^[^ ^3^ ^]^ All the above considerations mean that there is a profound need for an investigational glomerular model. This could be used both to improve understanding of the structure in health/disease, enhancing the development of therapies aimed at its repair, and to facilitate the drug development processes in general.^[^ ^2^, ^4^ ^]^

The majority of our understanding of glomerular cell physiology has come from either in vivo studies or from excision of the individual cell types followed by their culture in two‐dimensions in vitro. Whilst in vitro culture has provided a wealth of essential information the biological relevance of this is becoming more widely questioned and it does not reflect the gamut of complex cellular and other interactions that takes place in vivo. Taking a cell from a complex environment and placing it into a highly simplified system in culture will lead to inevitable losses in its natural biology. Enhancements can, however, be made. Providing more physiological growth conditions, such as a relevant basement matrix,^[^ ^5^ ^]^ improves the overall condition of the cells but is still a far cry from its normal situation. The addition of flow provides a further enhancement toward maturity and leads to alignment of the cell cytoskeleton, as observed in situ.^[^ ^6^ ^]^ In the past podocytes have been more troublesome to grow, but their generation from human induced pluripotent stem cells (iPSCs) has been a leap forward.^[^ ^7^ ^]^ However, these cells still lack some of the features of a mature cell when cultured in two dimensions. Out with of the structural role of each cell type, there is an additional strong symbiotic relationship that provides survival signals, but also regulates and limits damaging processes such as inflammation or proliferation. This relationship is crucial to maintaining the health of the whole glomerulus and the individual cell types within it. Consequently, removal of one cell type from this equation leads to dysfunction of the others.^[^ ^8^ ^]^ Co‐culture of endothelial cells and podocytes has illustrated the important cross‐talk that takes place to preserve the phenotype of both cell types ^[^ ^9^ ^]^ and, additionally, limit damage after injury.

Organoids offer an additional alternative tool in the challenge of studying kidney biology. These self‐organizing structures derived from iPSCs produce their own matrix and differentiate into a wide range of renal cell types.^[^ ^10^ ^]^ Whilst podocytes are prominent in most of the published work there is a paucity of both glomerular endothelial cells and mesangial cells,^[^ ^11^ ^]^ meaning that glomerular structures are not formed. This severely hampers the use of these as a tool for the biological understanding of glomerular function, especially filtration.^[^ ^12^ ^]^

Recent advances in microphysiological systems (MPS), such as organ‐on‐a‐chip technology, have enabled the incorporation of flow, stretch and co‐culture in a single system, dramatically improving our capabilities to explore and understand biological mechanisms.^[^ ^7^ ^]^ Some work has been done to develop glomerular chips that mimic the glomerular filtration barrier through the co‐culture of endothelial cells and podocytes on either side of porous membranes,^[^ ^7^, ^13^, ^14^, ^15^ ^]^ on ECM‐based gel walls,^[^ ^16^ ^]^ or on an interior and exterior surface of alginate‐hollow fibers with micro‐concave topography knot.^[^ ^17^ ^]^ However, there is currently limited glomerular MPS model that incorporates mesangial cells.^[^ ^18^, ^19^ ^]^ These cells are positioned within the heart of the glomerular assembly and provide a core of stability, regulating the structure and the pressures within.^[^ ^20^ ^]^ The absence of mesangial cells in current systems means that there is a fundamental difference to the natural state. Additionally, the ability to investigate processes/diseases where mesangial cells play an essential role is lacking. Even in vivo models, due to the lack of specific markers for mesangial cells to generate knock‐out models, are limited in modeling diseases where mesangial cells play a pivotal role. This includes glomerulonephritides such as IgA nephropathy and lupus nephritis or glomerulosclerosis, such as in diabetic kidney disease where Kimmelstein Wilson nodules are formed.^[^ ^20^ ^]^ Although the ability of all three glomerular cells to spontaneously form a glomerular‐like structure has been reported,^[^ ^18^ ^]^ functional 3D models that mimic a glomerular barrier have not yet been developed. This would shed light on the intricate relationship between mesangial cells and other glomerular cells in health and disease.^[^ ^20^, ^21^ ^]^ How much incremental benefit each additional improvement in culture conditions provides above the basal state of 2D culture has also not yet been shown. In this paper, we describe the design and production of a MPS containing all three glomerular cell types in physiological juxtaposition and under flow. We have compared the incremental advances that each cell type brings to the phenotype of the others, showing that enhanced complexity reduces the “disease‐state” observed in 2D cultures. Additionally, we have determined the benefits to the integrity of the filtration barrier. Overall, we demonstrate that this tri‐culture “Glomerulus‐on‐a‐chip” provides an enhanced device for modelling in vivo physiology and a useful tool in the armament of drug development.

## 2 Results and Discussion

### 2.1 Building a Tri‐Culture Glomerular‐Chip (TGC)

**Figure 1:**
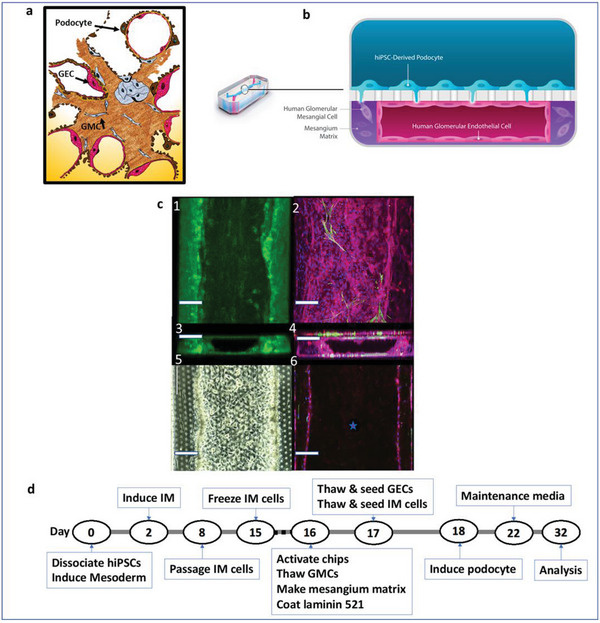
Physiological replication of the glomerulus in TGC. a) Schematic of a glomerulus showing essential elements and cell–cell and cell–matrix interactions. b) Schematic of the TGC. Transverse section through a TGC showing the juxtaposition of cells within the chip and matrix. c) Representative images of 1) acellular 3D fabricated mesangium and its lumen (top left; green, NHS‐Ester dye), 2) TGC (top right; αSMA in green, VE‐Cad in red, and nuclei in blue). Cross‐sections of the 3) acellular and 4) cell‐seeded chips, 5) light and 6) fluorescent microscope top views of cell‐seeded chips (bottom left and right, respectively). The star in 6 marks the lumen. Scale bars, 200 µm. d) Timeline of podocyte differentiation from hiPSCs in the TGC (intermediate mesoderm, IM; glomerular mesangial cell, GMC; glomerular endothelial cell, GEC).

**Figure 2:**
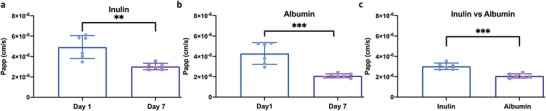
Albumin and inulin apparent permeabilities (P_app_) of TGC. a,b) Effect of culture time on barrier functions of TGCs (inulin a) and albumin b)). c) Comparison of permeability to insulin versus albumin in TGCs at 7 days. *n* = 6 independent chips (each dot on the graph represents the mean of triplicate measurements for an individual chip). Unpaired Mann‐Whitney test was used for all comparisons. **p < 0.01 and ***p < 0.001. Error bars represent mean ± SD.

**Figure 3:**
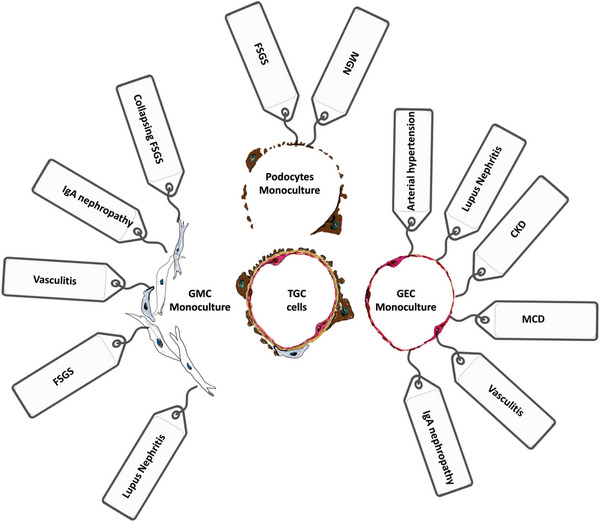
Pictorial representation of kidney diseases associated with pathways enriched in monocultures versus TGC. Analysis using Nephroseq highlighted disease associations of the enriched pathways (tags) for each of the three cell types. GECs, glomerular endothelial cells; GMCs, glomerular mesangial cells; TGC, tri‐culture glomerular chip; CKD, chronic kidney disease; MGN, membranous glomerulonephritis; MCD, minimal change disease; FSGS, focal segmental glomerulosclerosis.

**Figure 4:**
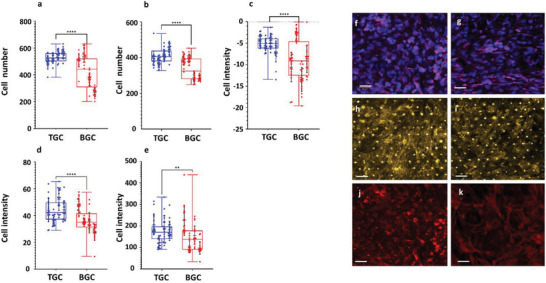
Automated medium‐throughput semi‐quantification of cell markers in tri‐culture and co‐culture chips. a) Podocyte cell number analysis in chips after differentiation (*n* = 4 independent chips with 18 selected areas per chip and total of 72 readings for each bar in graphs, each dot represents an individual reading). b) Cell number in the bottom of chips (total cell number for GMCs and GECs). c) Difference between total cell nephrin expression and membrane bound. d) Synaptopodin expression in podocytes in chips. e) EHD3 expression in GECs in chips. f,g) Representative images of nephrin expression (in magenta) in podocyte tri‐culture (f), co‐culture (g), (Dapi, DNA dye, is shown in blue). h,i) Representative images of synaptopodin expression (in gold) in podocyte tri‐culture (h) and co‐culture (i) chips. j,k) Representative images of EHD3 expression (in red) in GECs in tri‐culture (j) and co‐culture (k) chips. Scale bars in all, 50 µm. Unpaired Mann‐Whitney test was used for comparing two columns in each graph; Kruskal‐Wallis test and Dunn's multiple comparisons test were used for all comparisons in each graph. No significant difference, ns; **p < 0.01, and ****p < 0.001. Error bars represent mean ± SD.

**Figure 5:**
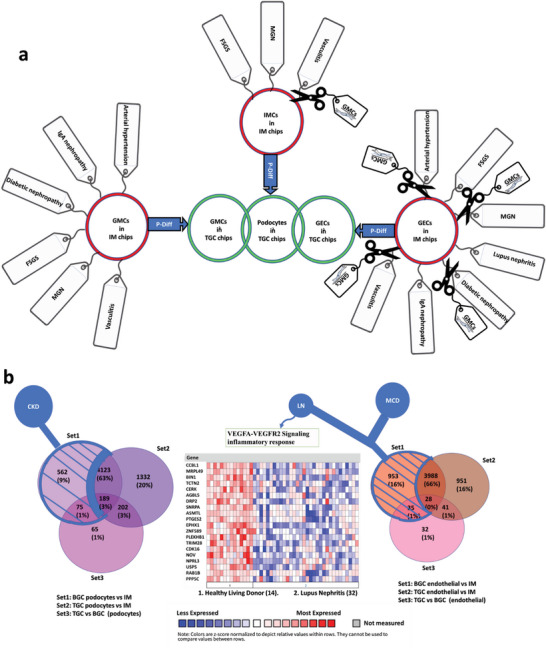
Influence of podocyte differentiation along with interplay of GMCs on gene signatures in TGC. a) Analysis of DEG in TGC containing either immature podocytes (IM) or fully mature podocytes. Nephroseq was used to compare DEG signatures in each cell type in each chip with human data from different kidney disease conditions. Associations were found in IM chips and diseases are shown in tags. Beneficial effects of GMCs on these associations are represented by scissor symbols. b) Left – Comparison of DEGs in podocytes cultured in BGC versus TGC with immature podocytes in chips, showing an association with CKD. Right ‐Comparison of DEGs in GECs in culture with immature podocytes or BGC versus TGC, showing an association with MCD and LN. Middle – Analysis of GECs in BGC versus TGC showed an enrichment for pathways associated with LN (Nephroseq). GMC, glomerular mesangial cells; GEC, glomerular endothelial cells; P‐Diff, podocyte differentiation; LN, lupus nephritis; MCD, minimal change disease; MGN, membranous glomerulonephritis; CKD, chronic kidney disease; BGC, co‐culture glomerular chip; TGC, tri‐culture glomerular chips; IM/C, intermediate mesoderm/cell.

To create our Glomerular‐Chip, we built on existing approaches^[^ ^22^ ^]^ using the Emulate S1 Chip. This Chip contains two central, parallel channels, both of which are under controllable unidirectional flow and are separated by a flexible porous membrane with a pore size of 7 µm. To incorporate the mesangial compartment, a physiologically relevant hydrogel (exhibiting both mechanical and compositional similarities to the human glomerular basement membrane) was incorporated into the bottom channel of the Chip to replicate the biological cell–cell/matrix interactions seen in vivo (**Figure** **1**a,b). The hydrogel was optimized to support cell growth and adhesion, whilst retaining tensile strength and supporting the generation of a lumen to be further seeded with endothelial cells (Figure 1c‐1; Video S1, Supporting Information). Mesangial cells within the gel stained positive for αSMA (Figure 1c‐2) and were responsive to serum and glucose concentrations (Figure S1, Supporting Information). In this investigation, both concentrations were adjusted to ensure cells were in a non‐proliferative state, as would be found in a healthy glomerulus (proliferation rate of 7.2% per week^[^ ^23^ ^]^). For future use, the adjustment of culture medium could provide an opportunity to mimic a more pathogenic pro‐proliferative environment, such as seen in the diabetic milieu. A lumen was created within the hydrogel to enable addition of endothelial cells. The endothelial cells adhered well and covered the entire surface (Figure 1c‐5,6; Videos S2 and S3, Supporting Information), showing a morphology reminiscent of that seen in situ. For a more comprehensive visualization of the arrangement and placement of mesangial cells within the hydrogel matrix in conjunction with endothelial cells, see Supplementary Videos (Videos S1–S3). Staining for the junctional marker VE‐cadherin exhibited a pattern demonstrating good cell–cell contacts (Figure 1c‐6).

In addition, we adapted and modified the previous protocol for deriving podocytes from iPSCs ^[^ ^22^ ^]^ which included modification of the protocol to include the final differentiation stage of the cells within the chip under static conditions. This led to reduced cell death and delamination (Figure S2‐1,2, Supporting Information). Staining for established podocyte markers illustrated a well‐defined actin cytoskeleton with synaptopodin distribution as expected and podocin staining showing membranes evocative of interdigitating foot processes (Figure S2‐3,4, Supporting Information).

### 2.2 The TGC Shows Improved Selectivity in Barrier Function

The fundamental role of the glomerulus is to restrict the loss of proteins from the circulation into the urine whilst allowing the passage of smaller waste molecules. Any model of this structure must fulfill those criteria to be at all useful. We therefore measured the passage of albumin and inulin as these are used in vivo as measures of glomerular barrier integrity, recognizing that the barrier should be freely permeable to inulin whilst constraining albumin. It is worth mentioning that the large membrane pore size of 7 µm is unlikely to affect the transport of albumin or inulin. The natural pore size of the glomerular barrier is about 4 nm. This would affect the filtration rate of proteins like albumin which has an ellipsoid shape with large and small diameters of 14 and 3.8 nm, respectively.

A time course showed that by day 7 a nadir in permeability to both inulin and albumin was reached with the restriction of albumin being greater than inulin as expected (**Figure** **2**). This change was not due to an increase in number of the barrier‐forming cells within the chip but likely due to the formation of better junctions (Figure S3, Supporting Information).

In healthy humans, an amount of albumin is still able to cross the filtration barrier (although we acknowledge that this area is contentious and much debate rages between experts). Estimates vary but a sieving co‐efficient of around 4×10^−4^ is suggested^[^ ^24^ ^]^, equating to about 3.6 g a day. This is then absorbed by the proximal tubules leading to no detectable albuminuria. This is demonstrated in those patients with Fanconi Syndrome who are unable to perform this function. Also, work^[^ ^25^ ^]^ looking at isolated human glomeruli has shown a permeability to albumin of ≈1×10^−6^ cm S^−1^. Based on our observations on day 7, we found that the albumin permeability in our model is very similar to that reported in previous studies. As such, we anticipated that a small amount of albumin would be able to pass through the barrier in our model, and we consider this to be a physiologically relevant mechanism. Under normal physiological conditions, the rate of albumin filtration is primarily dictated by the sieving coefficient of the glomerular filter rather than the concentration of albumin in serum. This underscores the importance, especially when comparing different platforms, of establishing an effective sieving coefficient. Doing so ensures that the rate of albumin filtration remains constant regardless of the specific platform employed. This approach significantly enhances the potential for biomimetic scaling of a given system and improves its capacity to accurately predict human clinical values.

Furthermore, it is essential to appreciate the intrinsic variability in albumin concentration across in vivo contexts. In the human body, albumin is not a solitary entity; rather, it exists in complex interplay with an array of other molecules, including bilirubin, ions, fatty acids, hormones, hemoglobin, and more. Each of these factors contributes to the overall permeability coefficient of albumin. Additionally, it is worth noting that our medium, like many in vitro systems, does not encompass cellular components, which play a crucial role in the determination of bioavailable albumin concentration and rheological properties.^[^ ^26^ ^]^

Our model's prediction of inulin clearance is similar to normal human kidney clearance, which ranges between 117–125 mL min^−1^ (3.51–3.75 µl h^−1^ for each nephron ^[^ ^27^, ^28^ ^]^). Our results show no significant difference in inulin clearance at the two timepoints (Figure S4a, Supporting Information). The discrepancy between clearance data and apparent permeability data can be attributed to the calculation methods. In addition, the decrease in inulin permeability may be due to a restriction that begins at a pore radius of 100 Å.^[^ ^29^ ^]^ Studies suggest this decrease in inulin permeability indicates improved barrier functions and integrity through the enhancement of tight junctions, as seen with increased occludin expression and redistribution and decreased MLCK and MLC phosphorylation.^[^ ^30^ ^]^

To examine the influence of the addition of mesangial cells on the barrier function of chips, we conducted a comparison between the barrier function of TGCs and BGCs in terms of the apparent permeabilities of inulin and albumin. The results revealed a significant reduction in albumin permeability in TGC groups as compared to the BGC groups, whereas no significant variation was observed in the passage of inulin between them (Figure S4b, Supporting Information).

The adjustment of flow rates and consequently shear stress within the channels did not alter permeability (Figure S5a–f, Supporting Information). We also observed that the effect of shear stress depended on the maturity of the podocytes (Figure S5a–f, Supporting Information). When cells were are mature and healthy, there was no significant change in the glomerular function (Figure S5c,d,f, Supporting Information), while for immature podocytes an increase in shear stress increased the permeability of albumin (Figure S5a,b,e, Supporting Information).

### 2.3 Complex Culture Systems Alter the Transcriptome of the Cells

It is widely accepted that culturing cells in different conditions will bring changes to their transcriptome. How extensive this is and what those changes are has not been well defined. We chose to examine this in our system in an incremental manner and to delineate the starting point of the cells in their basic 2D culture. This is important as it is the state that the vast majority of published research assumes as basal and healthy.

We took the top 1000 differentially expressed genes (DEGs) from each culture condition and compared them with human datasets in the publicly available platform of Nephroseq (www.nephroseq.org). We then performed pathway analysis to interrogate these findings (**Table** **1**). The 20 top associated genes were further investigated using gene ontology (MSigDB Biological Process).

**Table 1:** Associated genes and pathways (from gene ontology collection MSigDB Biological Process), and their P values for each comparison between TGC and less complex comparator systems (i.e., monocultures and co‐culture glomerular chips (BGCs)). P values are expressed for pathways and demonstrates extent of regulation.

Comparison	Cell type	Pathway	*P* value	Gene(s)
TGC vs monoculture	Podocytes	Primary FSGS	1.54e‐03	*PODXL, MYO1E*, and *COL4A4*
		PI3K‐Akt signaling pathway	2.87e‐02	*COL4A4*
		Focal adhesion	3.44e‐02	*COL4A4*
		Focal Adhesion‐PI3K‐Akt‐mTOR‐signaling pathway	3.62e‐03	*COL4A4*
		Apoptotic process pathway	1.00e‐09	*PDK4*
		Regulation of cell death	4.10e‐10	*PDK4*
		Response to oxygen‐containing compound	1.50e‐06	*PDK4* and PER
		Biological adhesion	< 1.00e‐32	*ITGB1* and *PODXL*
		Cell motility	2.20e‐11	*PODXL* and *MMP28*
		Cytoskeleton organization	4.40e‐11	*MYO1C, MYO1B*, and *MMP28*
		Cell junction organization	4.70e‐07	*MYO1C*
		Extracellular structure organization	6.50e‐12	*MYO1E, MMP28*, and *HTRA1*
	Mesangial cells	PI3K‐Akt Signaling	9.00e‐05	*ITGB1*
		Focal Adhesion‐PI3K‐Akt‐mTOR‐Signaling	6.40e‐06	*ITGB1*
		Regulation of cell population proliferation	≤ 1.00e‐32	*PHLDA2* and *ITGB1*
		Biological adhesion	≤ 1.00e‐32	*ITGA1, ITGB1*, and *EGFR*
		Negative regulation of inflammatory response	2.01e‐02	*PPARG*
		Inflammatory response	2.45e‐03	*EGFR* and *IFI16*
		Negative regulation of response to stimulus	≤ 1.00e‐32	*PPARG, ITGB1*, and *ITGA1*
		Regulation of response to external stimulus	8.70e‐05	*PPARG*
		Regulation of response to stress	2.50e‐04	*PPARG*
		Cytokine production	4.60e‐04	*IFI16*
		Apoptotic process pathway	6.00e‐10	*IFI16, XAF1, PHLDA2, LGALS1, PDK4*, and *EGFR*
		Regulation of intracellular signal transduction	2.00e‐11	*EGFR, LGALS1*, and *PDGFB*
		Tube morphogenesis	1.20e‐12	*UNC5B, PDGFRB, PDGFRA*, and *PGF*
		Extracellular structure organization	2.30e‐14	*ITGB1*
		Collagen catabolic process	3.90e‐04	*ITGB1*
		VEGF signaling pathway	1.00e‐04	*PDGFRB, PDGFRA*, and *PGF*
		VEGFR signaling pathway	1.85e‐03	*PGF*
	Endothelial Cells	Apoptotic process	9.80e‐06	*TSC22D3, IFI16, XAF1*, and *NOTCH1*
		Regulation of cell death	3.80e‐06	*TSC22D3, IFI16, XAF1*, and *NOTCH1*
		Response to osmotic stress	4.09e‐02	*TSC22D3* and *AQP1*
		Negative regulation of immune system process	3.05e‐03	*TSC22D3*
		Transforming growth factor beta receptor	2.40e‐04	*TGFBR1*
		Response to TGF‐β	7.30e‐08	*TGFBR1* and *HYAL2*
		Cellular response to VEGF stimulus	9.60e‐05	*NOTCH1, VEGFA*, and *VEGFC*
		Responses to oxidative stress	1.38e‐02	*AQP1* and *HYAL2*
		Response to retinoic acid	2.80e‐06	*AQP1*
		Responses to oxygen containing compound	5.40e‐09	*PDK4, AQP1*, and *HYAL2*
		Biological adhesion	8.40e‐12	*ICAM2, NOTCH1*, and *VEGFA*
		Taxis	2.20e‐11	*MATN2, NOTCH1, VEGFA*, and *VEGFC*
		Regulation of MAP kinase activity	1.70e‐05	*TGFBR1, HYAL2*, and *VEGFA*
		Aminoglycan catabolic processes	1.30e‐07	*HYAL2*
		Carbohydrate derivative catabolic processes	6.10e‐06	*HYAL2*
		Hyaluronan catabolic processes	2.71e‐02	*HYAL2*
		Cytoskeleton organization	3.40e‐11	*AQP1* and *GMFG*
		Glucose metabolic process pathway	5.10e‐04	*PDK4*
		Regulation of hydrolase activity	9.20e‐08	*IFI16*
		Negative regulation of response to stimulus	8.40e‐12	*NOTCH1*
		Response to growth factors	1.80e‐11	*HYAL2*
		VEGFR signaling pathway	1.86e‐02	*VEGFA* and *VEGFC*
		VEGF signaling	1.30e‐05	*VEGFA* and *VEGFC*
		Inflammatory response	1.51e‐03	*NOTCH1*
TGC vs BGC	Podocytes	EGFR	1.49e‐02	*KIF16B, ERBIN*, and *PTPN11*
		VEGF	3.96e‐02	*ROCK1, DOCK1*, and *EPN2*
		VEGFA‐VEGFR2 Signaling Pathway	4.20e‐05	*ROCK1, ITGB1, PTMA, PRKCI, PTPN11*, and *DHX36*
		Apical junction assembly	9.14e‐03	*ROCK1, TJP1, APC*, and *PRKCI*
		Cell projection assembly	1.20e‐09	*ROCK1, CD2AP*, and *ITGB1*
		Cell projection organization	6.90e‐05	*ROCK1, ITGB1*, and *PTPN11*
		Cell division	1.70e‐06	*PDS5B, CD2AP, USP16*, and *PDS5B*
		Cell cycle	1.40e‐09	*CD2AP, PDS5B, USP16, CDK6, TAOK1, USP47*, and *ITGB1*
		Mitotic cell cycle	2.90e‐05	*PDS5B, ITGB1, PTPN11*, and *USP16*
		Cellular response to DNA damage stimulus	1.30e‐10	*PDS5B, PTPN11*, and *USP16*
		DNA metabolic process pathway	7.50e‐08	*PDS5B, USP47, SETX*, and *TAOK1*
		RNA metabolic process	3.00e‐07	*ZC3H13* and *SETX*
		Regulation of protein modification process	4.88e‐02	*ROCK1, TAOK1*, and *PTPN11*
		Cellular protein metabolic process	8.30e‐05	*USP1, TAOK1, USP47, PTPN11, PDK4*, and *USP16*
		Cellular protein modification process	1.30e‐05	*TSPYL2, TAOK1, PTPN11, ROCK1, PDK4*, and *USP16*
	Endothelial cells	VEGFR signaling pathway	1.70e‐02	*ITGA5* and *FZD4*
		EGFR	8.36e‐03	*TGFB1*
		TGF‐βR	1.70e‐04	*TGFB1, TGFBR3, ITGA8, SMAD5*, and *SMAD7*
		Response to growth factor	8.70e‐04	*TGFBR3, ITGA5, FZD4, TGFB1, HYAL2, ITGA8*, and *SMAD7*
		Regulation of cellular response to growth factor stimulus	3.30e‐04	*TGFBR3, TGFB1, ITGA5, SMAD7*, and *FZD4*
		Response to TGF‐β	2.60e‐05	*TGFB1, TGFBR3, ITGA8, SMAD5, PDGFD*, and *HYAL2*
		Regulation of cellular response to TGF‐β	7.90e‐04	*TGFBR3, TGFB1, ITGA8*, and *SMAD7*
		Response to cytokine	4.07e‐02	*CD58, IFI16, ITGB1, TGFB1, SMAD7, HYAL2*, and *FZD4*
		Biological adhesion	2.20e‐04	*CD58, ITGB1, TGFB1, ITGA5, SMAD7, SLK, ITGA8*, and *FZD4*
		Cadherin binding	4.80e‐05	*SLK* and *ITGB1*
		Cytoskeleton organization	2.20e‐05	*TGFB1, ITGB1*, and *SLK*
		Actin filament‐based process	1.40e‐04	*TGFB1* and *ITGB1*
		Extracellular structure organization	4.00e‐03	*TGFB1, ITGB1, ITGA5*, and *ITGA8*
		Collagen catabolic process	1.41e‐03	*ITGB1*
		Regulation of hydrolase activity	1.24e‐02	*IFI16*,
		Positive regulation of catalytic activity	3.01e‐02	*SLK, FZD4, IFI16*, and *PDGFD*
		Response to oxygen containing compound	3.13e‐02	*TGFB1, HYAL2, TGFBR3, FZD4*, and *PDGFD*

Podocytes in monoculture displayed a gene signature demonstrating alterations to PI3K‐AKT signaling, cell death mechanisms, adhesion, motility, tight junctions, and importantly cytoskeleton. The absence of these gene expression patterns in the TGC points to increased adhesion, retention, and maturity in these podocytes. Two genes were significantly downregulated in monocultures; *PDK4* and *COL4A4*. These genes are important for metabolism and glomerular basement membrane production respectively and their expression in a less physiologically relevant manner suggests perturbations to these processes in 2D culture. Mesangial cells in monoculture showed differences in their inflammatory and proliferative phenotype compared to those in the TGC. They had greater differences in their pathways modulating extracellular matrix remodeling (e.g., PDGF pathways) and also metabolism (e.g., PPARG). Of note, the TGC mesangial cells increased activity of the VEGF pathway, potentially indicating a crosstalk with GECs to influence and support their maturation and vessel integrity. GECs in monoculture showed regulation of stress and cell death pathways, portending a less healthy phenotype. Modulation of the TGFβ pathway in the monoculture suggested that pathways involving endothelial‐mesenchymal transition may be active. *HYAL2* was significantly changed. This gene is involved in the formation of the glycocalyx structure indicating that these cells were attempting to construct this important component.

To establish the impact of the incremental addition of mesangial cells to our system, we compared it with a co‐culture chip (BGC) which included podocytes in the parenchymal channel and GECs within an acellular hydrogel in the vascular channel. The most notable differences between the tri‐culture and co‐culture chips involved pathways related to VEGF signaling, cell junctions, and actin cytoskeleton. Podocytes without mesangial cells appeared to be undergoing more cell cycle processes, but when mesangial cells were present, they increased their VEGF‐related signal which would be beneficial for the surrounding GECs. GECs showed modulation of a number of components in the TGFβ pathway, perhaps suggesting less endothelial to mesenchymal transition. Additionally, GMCs increased expression of genes involved in matrix modulation in GECs, making them more active in remodeling of the basement membrane.^[^ ^31^ ^]^ The overall impression was that the addition of mesangial cells improved the maturity and structure of all component cells.

Data from chips containing intermediate mesoderm cells (podocytes prior to full differentiation (see supplementary section for more details)) or mature podocytes were compared to determine how the maturation process affected other cell types present. Pathways modulated here predominantly involved renal vascular health (AngPT1, VEGF), immune mechanisms (IFNγ, IL8, TNF) or mesangial growth (TGFβ). This illustrates that mature podocytes influence the maturity of the surrounding cells. Within our data we were also able to investigate pathways involved in the podocyte differentiation process itself. Many of the pathways observed whilst comparing intermediate mesoderm to fully mature podocytes were replicative of those known to be involved in vivo; such as retinoic acid signaling and TGFβ, confirming our assumption that the process was biologically relevant (Table S1 and Figure S6a,b, Supporting Information). Particularly, we observed transcription of genes identified as markers of late podocyte progenitors such as Col4A4 and VEGFA[32] making this model also applicable for the study of glomerular differentiation and maturation.

Final analyses examined how mesangial cells influenced the differentiation of podocytes. It was apparent that the presence of mesangial cells improved the post‐mitotic characteristics of the cells, especially cytoskeletal health and lipid metabolic processes (Table S1, Supporting Information). Improvements in cytoskeletal health could indicate an influence of mesangial cells on the generation of foot processes in the podocytes, a finding never established previously. For GEC there were clear signals in pathways promoting health, notably that of VEGF. We believe that this is also a novel finding that the mesangial cells directly influence this.

### 2.4 Monoculture Gene Expression Patterns Show Signatures Reflective of Disease Phenotypes

Basic monocultures are the workhorse of cell research and basal culture conditions are deemed to be ground truth.^[^ ^33^ ^]^ We compared our top differentially regulated genes from each monoculture with gene sets from human renal disease using public data in Nephroseq (**Figure** **3**; Figures S7 for TGC and S8 for BGC, Supporting Information). Each monoculture showed enrichment in genes associated with human disease processes in the kidney, for example for podocytes *ITGB1*, for GEC *ICAM2* and for mesangial cell *PPARG*. Several of the associated signatures were also enriched in the cells most affected in that disease process, e.g., podocytes were enriched for FSGS (focal segmental glomerulosclerosis) signatures, mesangial cells showed IgA nephropathy (IgAN) signatures and endothelial cells were associated with vasculitis. However, these signatures were not exclusive to those cell types, displaying that when one glomerular cell type is damaged in disease this can affect the others.

Our transcriptional data suggest the production of glomerular basement membrane proteins by both glomerular endothelial cells and podocytes.^[^ ^34^ ^]^ Pathway analyses show that the GO‐basement membrane pathway in podocytes cultured in TGCs has been upregulated compared with those cultured on plates (P value: 2.2e‐09). Upregulated basement membrane‐related genes including laminins (521 as is seen in healthy human glomeruli), collagen IV (α4, which is not usually produced in vitro, demonstrating the advanced state of our model), *HSPG2* (perlecan), *VTN*, and Nidogen (*NID1* and *NID2*) suggested the physiological production of glomerular basement membrane by the cells in TGCs. Similarly, in endothelial cells, ECM‐related pathways such as ECM proteoglycans (P value: 6.2 e‐8), and ECM organization (P value: ≤1e‐32) have been upregulated in TGCs. Upregulation of glomerular basement membrane genes such as laminins (α1, α2, C3, C1, α3, B2), collagen IV (α4, α1, α2), *NID2* in endothelial cells cultured in TGCs compared to plate culture also suggest the formation of a glomerular basement membrane in this group.

### 2.5 Improvements in Gene Transcription Lead to Improvements in Protein Markers

Whilst gene changes are a useful measure of cellular responses, an equally important measurement is phenotype, such as structure and expression of protein markers. We therefore performed automated medium‐throughput image analysis on our chips to determine cell number and protein expression/localization in the presence (TGC) or absence (BGC) of mesangial cells. Analysis of podocyte number showed that the number decreased significantly in BGCs indicating either cell death or detachment (**Figure** **4**a). In TGCs, the number remained fairly static after seeding. Cell number in the bottom channel however increased over time with the ratio of GMCs to GECs maintaining ≈4.7 after podocyte differentiation (Figure S3, Supporting Information).

Nephrin content, both within the podocytes and localized at the membrane, was assessed (Figure S9, Supporting Information). Nephrin is a vital component of the slit diaphragm of the podocyte and therefore biological localization of the protein to the membrane is of functional significance. We analyzed the ratio of membrane to total expression as a measure of cell maturity and showed that there was a significantly higher proportion of the nephrin localized to the membrane in the mature TGC (Figure 4c; Figure S3, Supporting Information). This increase is in line with the increase in barrier function seen in the permeability assays and is likely to have had a direct impact on functionality.

Synaptopodin was also increased in TGC (Figure 4d). This protein is responsible for maintaining the complex actin cytoskeleton needed for the formation of foot processes. The higher and less variable levels seen in the TGC indicate that the podocytes have achieved a greater state of maturity and were forming higher order structures.

Finally, EHD3, a marker of glomerular endothelial cells, was also increased in the TGC chips (Figure 4e). EHD3 is important for the formation of endothelial fenestrations.^[^ ^33^ ^]^ These structures are usually difficult to acquire in an in vitro setting, but clearly the TGC provides an environment more conducive to their formation.

### 2.6 Podocyte Differentiation and the Role of Mesangial Cells

Studying the processes involved in in vitro podocyte differentiation may provide valuable insights into mechanisms involved in glomerulogenesis and glomerular barrier development. There are also potential therapeutic implications, as increased understanding could be used to modulate glomerular injury caused by podocyte dedifferentiation.

To determine the effect of podocyte differentiation on glomerular cells, we compared the TGC containing immature podocytes (i.e., intermediate mesoderm (IM) cells) to the mature TGC. Pathway analysis was performed on each cell type between these two groups (Table S1, Supporting Information). In addition, comparison of top regulated genes with human gene signatures (Nephroseq) (as described in section 2.3) revealed the immature TGC differentially expressed mechanisms associated with disease, whereas, in contrast, the mature TGC had no such associations (**Figure** **5**). DEG analysis also showed the importance of podocyte differentiation on renal vasculature development and mesangial development (Table S1, Supporting Information). In mature TGCs, podocyte expression of angiopoietin‐1 was higher (Figure S10a, Supporting Information). This pathway is known to contribute to the developmental maturation and remodeling of glomerular vasculature. Additionally, podocytes increased expression of integrinα3 (*ITGA3*) which is needed for the development and formation of mesangium (Figure S10a, Supporting Information).

We showed in vitro podocyte differentiation utilized several established mechanisms, including regulation of phosphatidylinositol‐3 kinase^[^ ^35^ ^]^ (PI3K), response to retinoic acid^[^ ^36^ ^]^ (RA), and TGFβ^[^ ^37^, ^38^ ^]^, demonstrating that the process recapitulates that seen physiologically. Dysregulation of these pathways has been associated with several glomerular diseases, including FSGS (PI3K pathway^[^ ^39^ ^]^) and renal vasculitis (RA^[^ ^40^ ^]^ and TGFβ^[^ ^41^ ^]^), so studying this process may give us insight into how to restore functionality of damaged podocytes.

IM cells were highly enriched for cell division pathways in contrast with podocytes in the TGC that were enhanced with negative regulation of cell proliferation pathways. This points to a postmitotic state for podocytes in TGCs versus IM cells. We also found enrichment in EMT and TGFβ regulation of ECM pathways (Figure S6a,b, Supporting Information). This would indicate an ongoing remodeling of the ECM. Podocyte EMT is thought to play a role in early glomerular injury^[^ ^42^ ^]^ via TGFβ^[^ ^43^ ^]^ and PI3K^[^ ^44^ ^]^. Studies have shown that inhibition of both TGFβ^[^ ^45^, ^46^ ^]^ and PI3K^[^ ^44^ ^]^ can block glomerular injuries. Our model could be a good way to study this phenomenon.

We also showed that in vitro podocyte differentiation has positive impacts on both GECs and GMCs achieved through modulation of several established pathways including EGFR, VEGF, TNFα, and NF‐κB (Table S1, Supporting Information). Dysregulation of these pathways has been associated with several glomerular diseases, including IgA nephropathy^[^ ^47^, ^48^ ^]^ (VEGF and TNFα), lupus nephritis^[^ ^49^ ^]^ (EGFR, NF‐κB, and VEGF), diabetic nephropathy^[^ ^50^ ^]^ (VEGF), vasculitis^[^ ^51^ ^]^ (TNFα), membranous nephropathy^[^ ^47^, ^52^ ^]^ (NF‐κB). In mature TGCs, endothelial expression of endothelial stabilizing ANGPT1 was higher. In contrast in immature TGCs, higher expression of *vWF* (a marker of vascular injury and endothelial dysfunction), pro‐fibrotic connective tissue growth factor (*CTGF*), and *ANGPT2* (which is known to destabilize vasculature) lead to the conclusion that a less mature endothelial layer is present (Figure S10b, Supporting Information). Additionally, in immature TGCs, mesangial cells increased expression of proinflammatory and profibrotic signals such as *IL‐6*, *CTGF*, *CCL2*, *FN1*, and *TGFβ1* (Figure S10c, Supporting Information). This model therefore offers insights into the influence of healthy podocytes on total glomerular health and how restoring podocyte health improves the functional capabilities of the glomerular endothelium and mesangium.

## 3 Conclusion

Recapitulating the complex structure of the glomerulus in vitro has proved challenging. No one model has succeeded in uniting all relevant cell types in a structurally correct manner to reconstitute the complex cell–cell/matrix interactions, in combination with physiologically relevant flow. This has meant that detailed biological questions surrounding the subtleties of glomerular physiology have remained unanswered. The results shown here suggest that the TGC is a promising tool with which to investigate glomerular biology in vitro. We have been able to develop a microphysiological system that contains all of the applicable resident cell types in the renal glomerulus in an anatomically relevant position, which also recapitulates the fundamental glomerular functional characteristic of albumin permeability (within the range of an *ex vivo* human glomerulus[25]). This model improves on the existing technology and advances understanding of the biology of the interplay between the different glomerular cells.

In the past, several in vitro models have been developed to recapitulate the glomerulus, but there have been key gaps.^[^ ^2^ ^]^ The most fundamental of these has been the lack of mesangial cells within the structure. Mesangial cells play a key role in glomerular biology in health, removing deposits and lipids to maintain structural health as well as regulating haemodynamics and contributing to immune responses.^[^ ^20^ ^]^ Without the presence of this important cell type a glomerular model will not fully replicate the biology of in vivo situations.

Our primary analyses have investigated the differences our advanced in vitro technology conditions have made to each cell type. We have been able to demonstrate improvements to each of the cell types contained within the TGC. Podocytes in the TGC exhibited major improvements in structural composition, as demonstrated at a gene and protein level. Nephrin was preferentially localised at the cell borders, no doubt contributing to the improved retention of albumin, and the cytoskeletal structures were well defined with suggestions of foot formation. This was further confirmed within the gene signatures for this cell type. For GEC, there were well defined junctional connections, again reflected in the improved barrier function, and EHD3 staining suggested fenestrations could be formed. We were unable to perform electron microscopy to confirm this. For mesangial cells, it was evident that the TGC promoted a more quiescent phenotype, but more importantly it highlighted the crucial role that this cell type has in facilitating the healthy phenotype of the other two cell types.

One of the most important findings centers around the difference seen in 2D monocultures versus our co‐culture 3D system. We have demonstrated that single cultures often exhibit gene signatures that can be detected in human disease settings. For 2D podocytes, dysregulation of many of the detected pathways have been associated with several glomerular diseases, including FSGS^[^ ^39^ ^]^ (cell motility, apoptosis, and PI3K) and membranous glomerulopathy^[^ ^47^ ^]^ (cytoskeleton and apoptotic pathways). For mesangial cells inflammatory and proliferative changes have been reported in several glomerular diseases, including lupus nephritis^[^ ^21^, ^53^ ^]^, membranous glomerulopathy^[^ ^21^ ^]^, FSGS^[^ ^54^ ^]^, IgA nephropathy^[^ ^48^ ^]^, and renal vasculitis.^[^ ^55^, ^56^ ^]^ Finally for GEC stress and cell death have been associated with several glomerular diseases, including lupus nephritis,^[^ ^47^ ^]^ arterial hypertension[57], CKD^[1,58]^, renal vasculitis^[^ ^59^, ^60^ ^]^, minimal change disease^[^ ^47^ ^]^, and IgA nephropathy.^[^ ^61^ ^]^

Currently, simple in vitro models assume that cells are reflective of a healthy cell phenotype in vivo. We demonstrate here and previously ^[^ ^62^ ^]^ that this is far from certain and that even primary cells isolated from normal tissues, cannot be considered healthy if not cultured in their relevant physiologic microenvironments. This highlights that the presence of a cell's neighbors and the resulting paracrine cues are fundamental to its physiology and ignoring this reduces the translatability of research findings to the in vivo organism. Indeed, when using simple 2D systems we run the risk of merely changing cells from one dysfunctional state to another. Incremental addition of complexity to 2D models may ameliorate this to a greater or lesser extent and researchers should judge if the mechanism they are investigating is truly fully reflected in a healthy state in the system they are using.

There are obvious flaws in our system. GECs and podocytes are still separated by an artificial membrane whose properties are not reflective of the glomerular basement membrane. However, contacts were observed between the extensions of podocyte cell processes and GECs through pores in the membrane^[^ ^22^, ^42^ ^]^ and the pores are large enough for the passage of soluble mediators, the main protagonists of the cross‐talk in vivo. In addition, our system is technically complex for the untrained person and is low throughput. That being said, these are not insurmountable problems and could be engineered out.

The TGC presented here is a unique system. Its 3D structure provides necessary interactions, contact‐dependent (i.e., cell–cell and cell–matrix) and independent (via soluble mediators), between key players in the glomerular microenvironment, in a well‐defined microfluidic device where flow rates and mechanical forces (e.g., deformation, strain, and fluid shear stress) are controlled. The combination of engineering technology and biological concepts has provided an opportunity to investigate healthy physiology in this complex structure as well as the possibility to study diseases driven by changes to the mesangium, not previously possible. This opens the window to fully study conditions such as IgA nephropathy,^[^ ^47^ ^]^ and diabetic kidney disease^[^ ^63^ ^]^ where mesangial cells are known to have a pivotal role.

The ultimate prize would be to fully replicate renal biology by adding the rest of the nephron. It is possible to imagine a situation whereby a vascularized tubule containing appropriate stromal cells is added to this technology and linked to enable feedback to occur. Such advances would then permit the full gamut of renal physiology to be studied.

## 4 Experimental Section

### Materials

Primary human cells and PGP1 cells obtained from Cell Systems and the Personal Genome Project, respectively. Fetal bovine serum (FBS), phosphate‐buffered saline (PBS), DMEM/F12 with GlutaMax supplement, BMP7, B27, activin A, VEGF, laminin 521, TrypLE, Hoechst 33342, Phalloidin‐488, primary antibodies—including EHD3, PDGFR β, α‐SMA—and all secondary antibodies were purchased from Thermo Fisher Scientific. Other primary antibodies for immunofluorescent staining of podocytes (including Nephrin and Synaptopodin) and endothelial cells (VE‐cadherin) were acquired from Progen and Santa Cruz, respectively. Collagen IV (isolated from human placenta which is a mixture of COL4A1 isoform 2, COL4A2, COL4A3, and COL4A2 isoform 3), TRI Reagent, and all‐trans retinoic acid were obtained from Sigma. Collagen I and fibronectin were purchased from Advanced BioMatrix and Corning, respectively. CHIR99021 and ROCK inhibitor were obtained from Stemgent and Tocris, respectively. All reagents were used as received.

### Study Design

A random assignment of individual chips was performed within each experiment for all analyses. In barrier function studies, only chips featuring no flow issues were used to avoid any potential bias (6 chips per group). For automated medium‐throughput semi quantification, 18 individual designated spots were analysed on each chip covering the entire chip (a total of 72 spots for each experimental group). Cell numbers in each spot were in the range of 338–575 for the bottom channels and 418–555 cells for the top channels. All measurements were carried out at least in triplicate and the means of triplicate measures were used.

A series of optimizations were performed. 1) Incorporation of mesangial matrix into the bottom channel of the chips, including parameters for the viscous fingering technique (hydrostatic pressure, timing for gel formation, gel composition/concentration), chip activation protocols and cell seeding densities, 2) podocyte differentiation protocols (including the composition of ECM coating (laminin 521), thawing protocols for IM cells, time to start of flow in Zoë (Emulate microfluidic platform, Emulate Inc.), podocyte induction time and media), 3) immunofluorescence staining protocols of the TGCs, 4. barrier function studies. The repeatability and reproducibility of the optimized protocols for the fabrication of TGCs (Figure 1d) was tested and validated by several independent individuals

### Cell Culture

Primary human glomerular microvascular endothelial cells (Cell Systems), primary human GMCs (Cell Systems) and PGP1 (the Personal Genome Project) hiPSCs were all propagated according to the supplier's instructions using the recommended media and were mycoplasma negative. Using the same media for both channels offered advantages in studying transport and barrier functions, and it was ensured by carefully testing the recommended media. To avoid any possible changes in phenotype or genotype of the cells the same passage number for each cell type for all experiments was used.

### Differentiation of hiPSCs to Podocytes

A working cell bank of intermediate mesoderm cells was made according to the previously published protocol.^[^ ^22^ ^]^ The protocol for induction of podocytes was optimized in 48‐well plates (Falcon Clear Flat Bottom TC‐treated Cell Culture Plate). Briefly, plates were coated with laminin 521 (50 µg mL^−1^, Gibco) for 2 h at 37 °C. Intermediate mesoderm cells (calculated to reach a cell density of 100 000 cells well^−1^) were resuspended in induction media supplemented with ROCK inhibitor and 20% FBS (DMEM/F12 with GlutaMax supplement (Gibco), 100 ng mL^−1^ BMP7 (Gibco), 3 µm CHIR99021 (Stemgent), 1× B27 serum‐free supplement (Gibco), and 10 µm Y27632 ROCK inhibitor (Tocris). Cells were fed daily for 4 days with podocyte induction medium consisting of intermediate mesoderm induction media supplemented with 100 ng mL^−1^ activin A (Gibco), 50 ng mL^−1^ VEGF (Gibco), 3 µm and 0.1 µm all‐trans retinoic acid (Sigma). For chip experiments IM cells were directly seeded onto chips pre‐seeded with human glomerular microvascular endothelial and mesangial cells. IM cells were seeded at 4 000 000 cells mL^−1^, cultured under static conditions and medium was changed daily for 4 days.

### Preparation of Tri‐Culture Glomerular Chips

The top channel was coated with 50 µg mL^−1^ laminin 521. The bottom channel gel comprised DMEM/F12 media mixed with NaOH (0.125 µg mL^−1^), fibronectin (66 µg mL^−1^, Corning), collagen IV (66 µg mL^−1^, Sigma), collagen I (10 mg mL^−1^, FibriCol from Advanced BioMatrix). Intermediate mesoderm cell (podocyte) seeding density was optimized to cover the whole top channel co‐culture area and selected based on previously published protocols.^[^ ^22^, ^42^ ^]^ It was attempted to keep the endothelial and mesangial cell densities in chips the same as the human glomerulus^[^ ^64^ ^]^ whilst ensuring the endothelial cells covered the whole surface of the lumen to provide a functional barrier. To create a lumen within the hydrogel matrix, after introducing an ice‐cold ECM solution (20 µL) containing mesangial cells (at 220 000 cells chip^−1^) into the bottom channel, a 1 mL tip was placed containing 300 µL cold DMEM media in the inlet of the bottom channel to flow through gravity and static pressure and replace the ECM solution in the center of the endothelial channel, then chips were placed into the incubator to solidify the ECM. Primary human GECs (8.5 × 10^4^ cells chip^−1^) were seeded in complete medium with Rocket Fuel‐R plus 10% FBS by inverting the chip for 2–3 h to ensure complete coverage.

The day following endothelial seeding, the top channel was seeded with 16.6 × 10^4^ hiPSC‐derived IM cells per chip in the IM‐inducing medium described. Podocyte induction medium was introduced after achieving full coverage of IM cells on the top of membrane and refreshed daily for 4 days.

After differentiation of podocytes, chips were connected to a Zoë where a volumetric flow rate of 60 µL h^−1^ (shear stress of 0.0007 dyne cm^−2^ for the top channel and 0.136 dyne cm^−2^ for the bottom channel), was continuously applied. The addition of 3D structure in the glomerular chips resulted in an 8‐times increase in the shear stress compared with plain S1 Chips with no 3D hydrogel in the bottom channels (0.017 dyne cm^−2^) that is much closer to the shear stress in the glomerulus (0.7–1.2 dyne cm^−2^). The TGCs were cultured for 7 days before analyses. The five main groups of this study were mature (podocytes) and IM cells tri‐culture chips, co‐culture chips (only GECs and podocytes) and control IM seeded chips.

### Immunostaining and Image Analysis

Chips were fixed with 4% formaldehyde for 30 min, at room temperature before being washed and permeabilized (Triton X 0.1% with 2% BSA in PBS). Blocking and incubation with the primary antibodies (overnight in PBS containing 2% BSA) was followed by a 2 h incubation with fluorescently conjugated secondary antibodies (Invitrogen), Phalloidin‐488 (1:500, Thermo Fisher Scientific) and Hoechst 33342 (1:1000, Invitrogen). Immunostaining was performed with primary antibodies specific for the Synaptopodin (1:100) and Nephrin (1:50) for podocytes (61094 and GP‐N2 from Progen, respectively), VE‐cadherin (1:100, sc‐9989, Santa Cruz) and EHD3 (1:50, PA5‐14365, Thermo Fisher Scientific) for GECs, and PDGFR β (1:100, MA5‐15143, Thermo Fisher Scientific) and α‐SMA (1:100, MA5‐15143, Invitrogen) for GMCs.

To quantify the expression of proteins in chips for different groups, confocal fluorescent images were acquired using a Cell Voyager 7000 (CV7000, Yokogawa Inc.) and an automated scanning protocol according to previously published methods.^[^ ^65^ ^]^ Images (18 fields of view, spanning the length of each chip) were captured at 20× magnification over a 120 µm range at 5 µm Z intervals to cover both cell layers (i.e., podocyte layer and mesangial layer including GECs and GMCs). Analyses were performed on the maximum intensity projection image from each cell layer, for the same 18 fields of views on every chip. Cell stain intensities were calculated using Perkin Elmer Columbus software.

### Confocal Image Acquisition

Confocal images of the bottom and top layers of the chip were captured on an inverted Zeiss LSM880 confocal microscope equipped with Airyscan detector using Plan‐Apochromat 10×/0.45 M27 objective lens and 2× digital zoom. The chip was mounted on a thin coverslip with FluorSave mounting medium. Hoechst33342, α SMA, Synaptopodin, Nephrin, and EHD3 were imaged using 405, 488, 561, 633, and 633 nm excitation lasers, respectively. To cover both bottom and top channels, Z stack imaging was performed separately each with optimized Z intervals of 4.131 and 3.829 µm, respectively, for total thickness of 250 µm. The images were extracted with ZEN black software version 2.3 SP1.

### Inulin and Albumin Filtration Assay

To evaluate the integrity of the glomerular barrier, TGCs were subjected to fluid flow for 7 days. To obtain information regarding the maturation of the cells over time, filtration assays were performed at two time points (after differentiation and day 7). In these assays basal serum‐free media (i.e., the complete medium with Rocket fuel‐R) was perfused through both top and the bottom channels of the chips. For filtration assays, the medium in the bottom channel was supplemented with fluorescently labeled albumin and inulin (100 µg mL^−1^ human albumin conjugated with Texas Red, Rockland Antibodies & Assays and 10 µg mL^−1^ Fluorescein isothiocyanate‐inulin, Sigma). Based on estimates for osmotic pressure versus albumin concentrations, the change in osmotic pressure and consequently glomerular filtration resulting from the slight increase in albumin concentration was negligible.^[^ ^66^ ^]^ The concentrations used in the system do not fully reflect those either present in plasma (albumin, 45 g L^−1^) or those given when measuring GFR (inulin, 3–400 mg L^−1^) so direct comparisons are not possible, but choosing values similar to physiology has its limitations for MPSs and is not easily achievable. The standard protocols were chosen to be followed to have a better comparison of the model with previously published studies that took into account the size and surface area of the system when calculating values compared with the physiological values.^[^ ^22^, ^42^ ^]^ There was a wide range of concentrations used in this type of assay, and no “gold standard” had been established. Some researchers use higher concentrations,^[^ ^13^, ^16^, ^67^, ^68^ ^]^, while others employ the same concentration as we do.^[^ ^7^, ^15^, ^22^ ^]^ Even some leading experts in this field have adjusted their concentrations over time.^[^ ^69^, ^70^ ^]^ The lack of consistency in concentration choices was likely attributable to the numerous other parameters that influence filtration and permeability, including residence time, flow rate, assay duration, flow direction (unidirectional vs bidirectional), culture surface area, pressure, shear stress, and temperature. Some of these parameters had been addressed in the model development. The choice of albumin concentration at 100 µg mL^−1^ aligned with the objective of ensuring reproducibility across different studies and forging a path toward much‐needed standardization in the field.

All fluorescence intensities were measured after 24 h of continuous perfusion flow using a Synergy NEO HTS Multi‐Mode microplate reader (BioTek). The renal apparent permeabilities for both fluorescently labelled inulin and albumin (from the microvascular bottom channel to the top channel) were calculated based on the following equation:

(1) $$P_{\mathrm{app}}=-\frac{Q_{R}*Q_{D}}{SA*\left(Q_{R}+Q_{D}\right)}*\ln\left[1-\frac{C_{R,0}*\left(Q_{R}+Q_{D}\right)}{Q_{R}*C_{R,0}+Q_{D}*C_{D,0}}\right]$$

where Papp is the apparent permeability in units of cm S^−1^, SA is the surface area of the co‐culture channel (0.17 cm^2^), Q_R_ & Q_D_ are the fluid flow rates in the receiving and dosing channels, respectively, in units of cm^3^ S^−1^, and C_R,0_ & C_D,0_ are the recovered concentrations in the receiving and dosing channels, respectively, in any consistent units.

### Sample Preparation for RNA‐seq Analysis

Several experimental conditions were compared including chip groups (tri‐culture and co‐culture chips with intermediate mesoderm cells as well as differentiated mature podocytes on the top channel) and plate cultures (i.e., monocultures for glomerular endothelial cells, glomerular mesangial cells, intermediate mesoderm cells, and podocytes). For each condition, there were 4 replicates. For RNA preparation from monocultures each cell type was lysed separately in lysis reagent (TRI Reagent, Sigma). For chip experiments GECs were extracted using TrypLE (TrypLE, Gibco) and then lysed with TRI reagent. GMCs remained behind after this procedure and were isolated by injecting lysis reagent into the bottom channel and aspirating. For podocyte extraction, lysis reagent was injected into the top channel and aspirated. RNA extraction and RNA‐seq analysis was performed by Lexogen (Lexogen, Vienna, Austria). The number of replicates for each RNA‐seq analysis was 4.

### Whole‐Transcriptome Analysis (RNA seq)

RNA‐seq data was analyzed using Rosalind (https://rosalind.onramp.bio/). All gene enrichment analyses, differential expressions, pathway analyses on differentially expressed genes, and meta‐analyses was performed by Rosalind with a HyperScale architecture developed by OnRamp BioInformatics, Inc. (San Diego, CA). Comparison of the top regulated genes with healthy and diseased human biopsies was performed utilizing Nephroseq (https://www.nephroseq.com/). All information regarding the data processing including read trimming (by the cutadapt tool), alignment (to Genome Reference Consortium Human Build 38, by STAR tool), aggregated quality controls (e.g., read distribution statics and plots (counts) by the MULTIQC tool), and reproducibility are available upon requested.

### Statistical Analysis

One‐way analysis of variance (ANOVA) was performed followed by Tukey's post hoc multiple comparison tests with a single pooled variance and unpaired t tests (two tailed) assuming Gaussian distribution (parametric data), as well as Kruskal–Wallis tests, Dunn's multiple comparisons tests, and the Mann–Whitney tests for non‐parametric tests using Prism 9 (GraphPad). P values less than 0.05 were considered statistically significant.

## Conflict of Interest

L.E., V.K., and R.L. are employees or former employees of Emulate Inc. and may hold stock in the company. L.E., S.P., A.C., B.N., P.B.L.H., and J.W. are employees or former employees of AstraZeneca PLC and may hold stock in the company. The other authors declare that they have no competing interests

## Author Contributions

R.P. and J.W. conceived the study and designed the experiments. R.P., R.L., V.K., and H.W. performed all experimental work in this study. J.W. and L.E. supervised the team. S.P. and A.C. performed automated medium‐throughput analysis of protein expressions. R.P. performed RNA‐seq data analysis and statistics. B.N. performed confocal microscopy. R.P. performed the data analysis and statistics. R.P. and J.W. wrote the manuscript, L.E. performed a critical review. R.P. prepared illustrations and figures.

## Supporting information

Supporting Information

Supplemental Video 1

Supplemental Video 2

Supplemental Video 3

## Data Availability

The data that support the findings of this study are available from the corresponding author upon reasonable request.
