# Supplementary material for: Physiological Replication of the Human Glomerulus Using a Triple Culture Microphysiological System
Source: Adv Sci (Weinh). 2023 Oct 22;10(33):2303131. doi: 10.1002/advs.202303131 (PMC10667800; doi:10.1002/advs.202303131)
Supplement: Supplementary file 1 — Supporting Information [file ADVS-10-2303131-s004.pdf]

ADVANCED SCIENCE
Open Access

Supporting Information

for *Adv. Sci.*, DOI 10.1002/advs.202303131

Physiological Replication of the Human Glomerulus Using a Triple Culture Microphysiological System

*Ramin Pajoumshariati, Lorna Ewart, Ville Kujala, Raymond Luc, Samantha Peel, Adam Corrigan, Heather Weber, Bramasta Nugraha, Pernille B. L. Hansen and Julie Williams**

Supplementary Materials for
Physiological replication of the human glomerulus using a triple culture microphysiological system.

Ramin Pajoumshariati[1], Lorna Ewart[2], Ville Kujala[2], Raymond Luc[2], Samantha Peel[3], Adam Corrigan[3], Heather Weber[1], Bramasta Nugraha[1], Pernille B L Hansen[1], Julie Williams[1*]

[1]Bioscience Renal, Research and Early Development, Cardiovascular, Renal and Metabolism (CVRM), BioPharmaceuticals R&D, AstraZeneca, Gothenburg, Sweden; [2]Emulate Inc., Boston, MA., United States; [3]Functional Genomics, Research and Early Development, Discovery Sciences, BioPharmaceuticals R&D, AstraZeneca, Cambridge, UK. [4]Vertex Pharmaceuticals, Boston, MA., United States. *e-mail: Juliem.williams@astrazeneca.com

This PDF file includes:

Supplementary Figures S1–10

Table S1

Captions for Videos S1–3

Other Supplementary Materials for this manuscript includes:

Videos S1–3

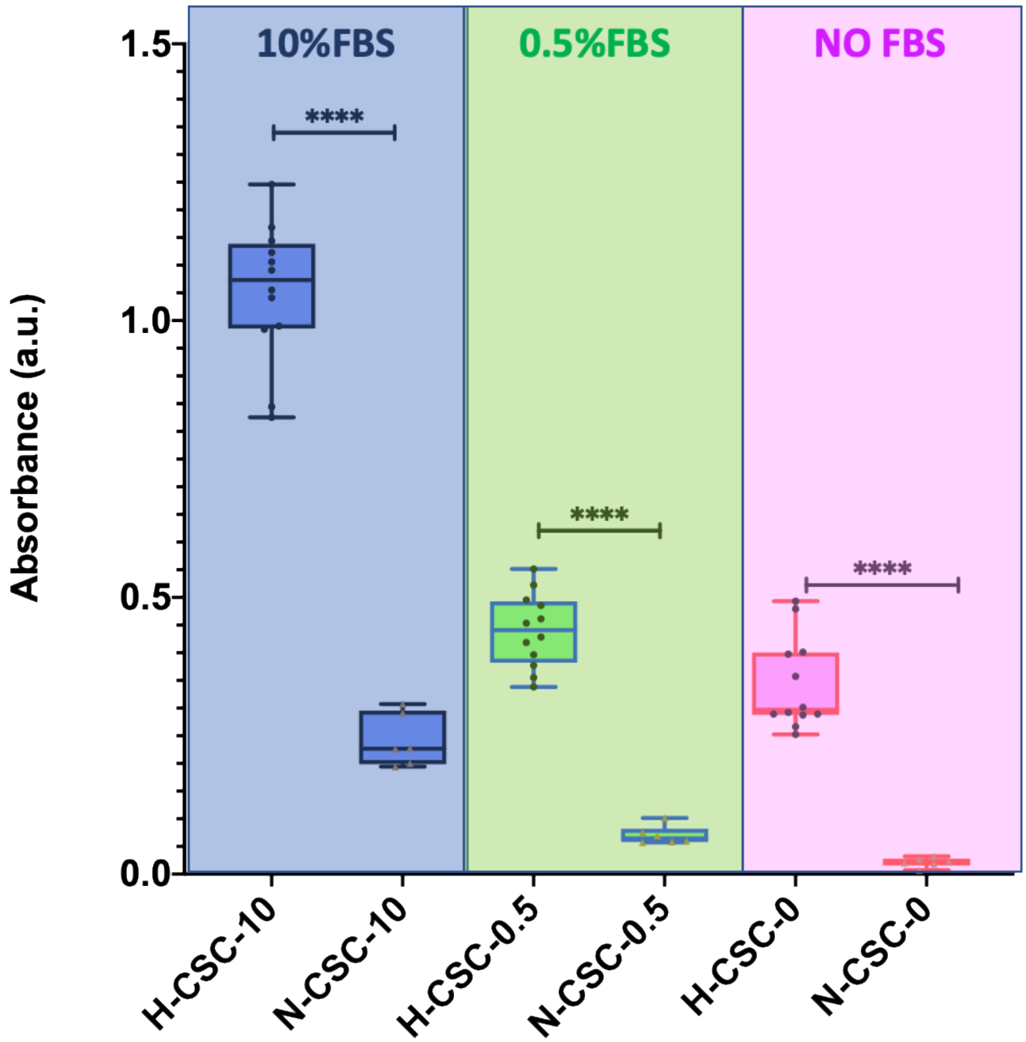

Figure S1. Effect of serum and glucose concentrations. The proliferation of GMCs in 3D fabricated mesangium at different glucose and serum concentrations (higher glucose level, H-CSC; normal glucose, N-CSC; numbers in front of the media denote serum concentration percentages (i.e., 10, 0.5, and 0%)). n= at least 6 independent chips for each group. Unpaired Mann-Whitney test was used for all comparisons. ****$P < 0.0001$. Error bars present mean ± SD.

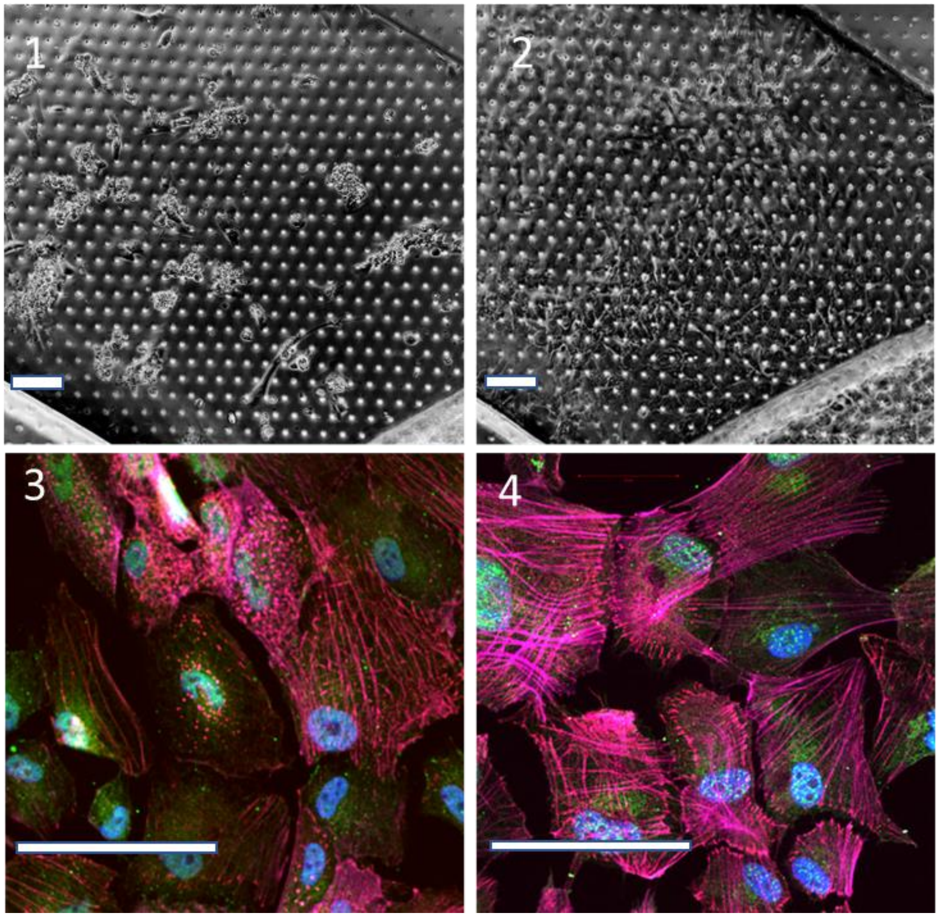

Figure S2. Representative images of podocytes after differentiation in glomerular chips in absence (1&3) and presence (2&4) of continuous flow. The images were captured at the outlet of the top channel, specifically in an area where only podocytes are located (i.e., not a co-culture area) on a non-porous surface and are thus more sparsely populated than within the channel itself where imaging is more difficult. 1 and 2 are light microscopic images. 3 and 4 are immunofluorescence images of podocytes (podocin in green, synaptopodin in red, actin in magenta and DNA in blue) showing the disrupted synaptopodin and actin fibres in podocytes using the unmodified protocol (bottom left, 3) versus healthy podocyte cytoskeleton (bottom right, 4). Scale bars, 100 µm.

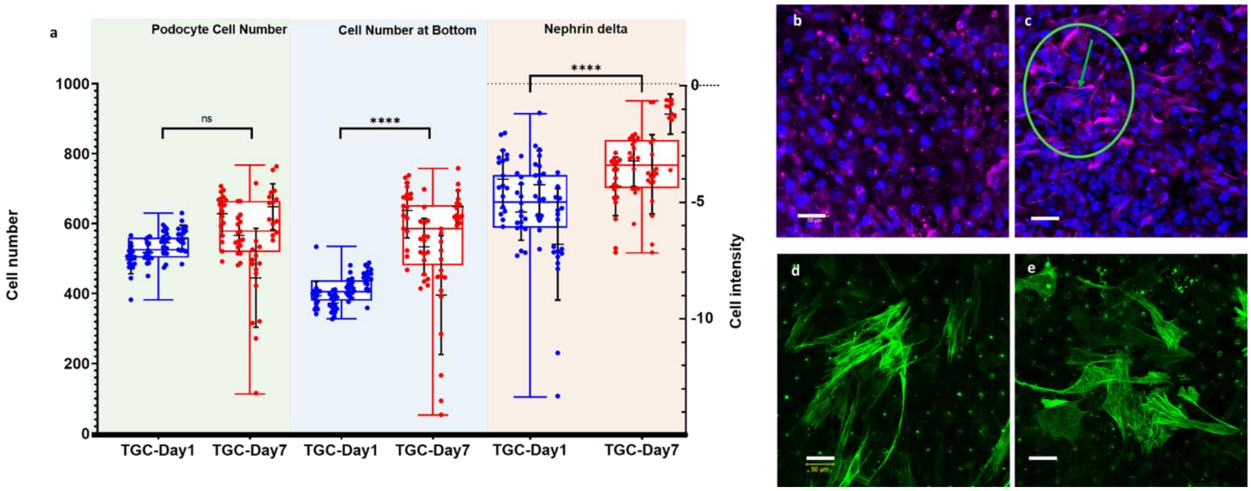

Figure S3. High throughput quantification inquest of tri-culture chip maturation over time. a) Podocyte cell number analysis, cell number in the bottom of chips (total cell number for GMCs and GECs), and difference between total cell nephrin expression and membrane bound. (n=4 independent chips with 18 selected areas per chip and total of 72 readings for each bar in graphs). b,c) Representative images of nephrin expression (in magenta) in podocyte tri-culture at day 1(b) and day 7 (c), (Dapi, DNA dye, is shown in blue,) (in c the arrow is pointing to the membrane localization of nephrin). d,e) Representative images of αSMA expression (in green) in GMCs in tri-culture at day 1 (d) and day 7 (e). (Scale bars in all, 50 µm). Unpaired Mann-Whitney test was used for comparing two columns in each graph; Kruskal-Wallis test and Dunn's multiple comparisons test were used for all comparisons in each graph. No significant difference, ns; and ****$P < 0.001$. Error bars represent mean ± SD.

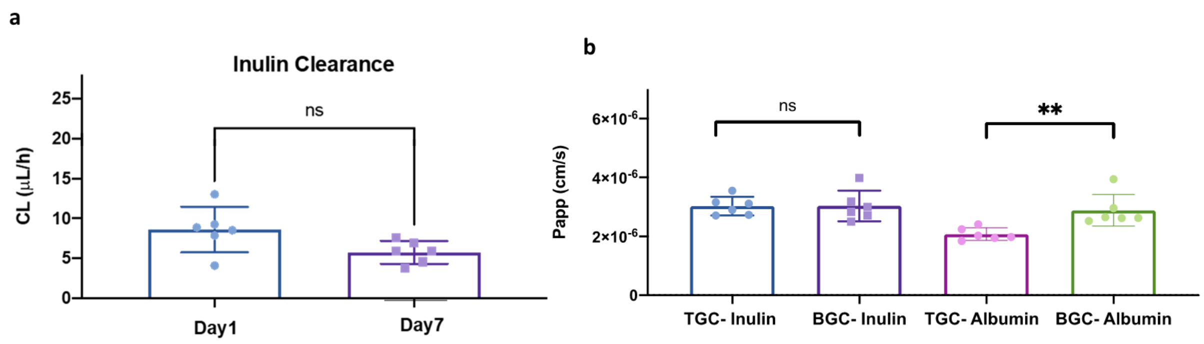

Figure S4. a) Inulin clearance (CL) of TGC. Effect of culture time on inulin clearance. b) Comparison between barrier function in TGCs versus BGCs. n=6 independent chips (each dot on the graph represents the mean of triplicate measurements for an individual chip). Unpaired Mann-Whitney test was used for all comparisons. No significant difference, ns; and ***P* < 0.01. Error bars represent mean ± SD.

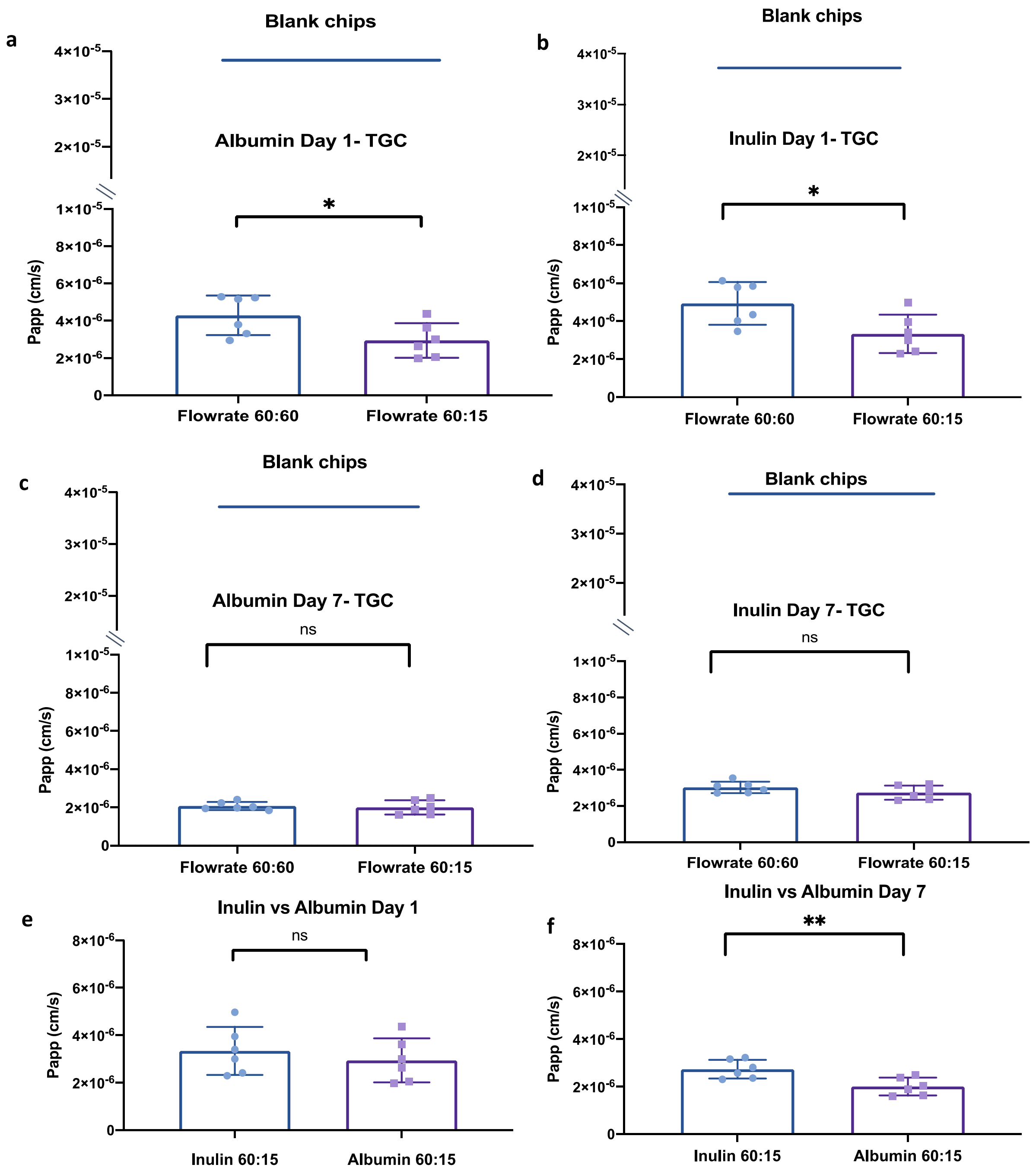

Figure S5. Effect of flow rate's adjustment on barrier function of tri-culture glomerular chips represented by albumin and inulin apparent permeabilities (P_{app}). a–d) Effect of top channel flowrate (60 vs 15 µL h^{-1}) on barrier functions of TGCs at day1 (albumin (a) and inulin (b)) and day 7 (albumin (c) and inulin (d)). e,f) Selective transport of molecules in TGCs (top channel flowrate: 15 µL h^{-1}) at day 1 after differentiation (e) and day 7 (f). numbers in front of the X axis groups denote "bottom : top" flowrates (µL h^{-1}). n=6 independent chips with triplicate measurement for each point. Unpaired Mann-Whitney test was used for all comparisons. No significant difference, ns; *$P < 0.05$ and **$P < 0.01$. Error bars present mean ± SD.

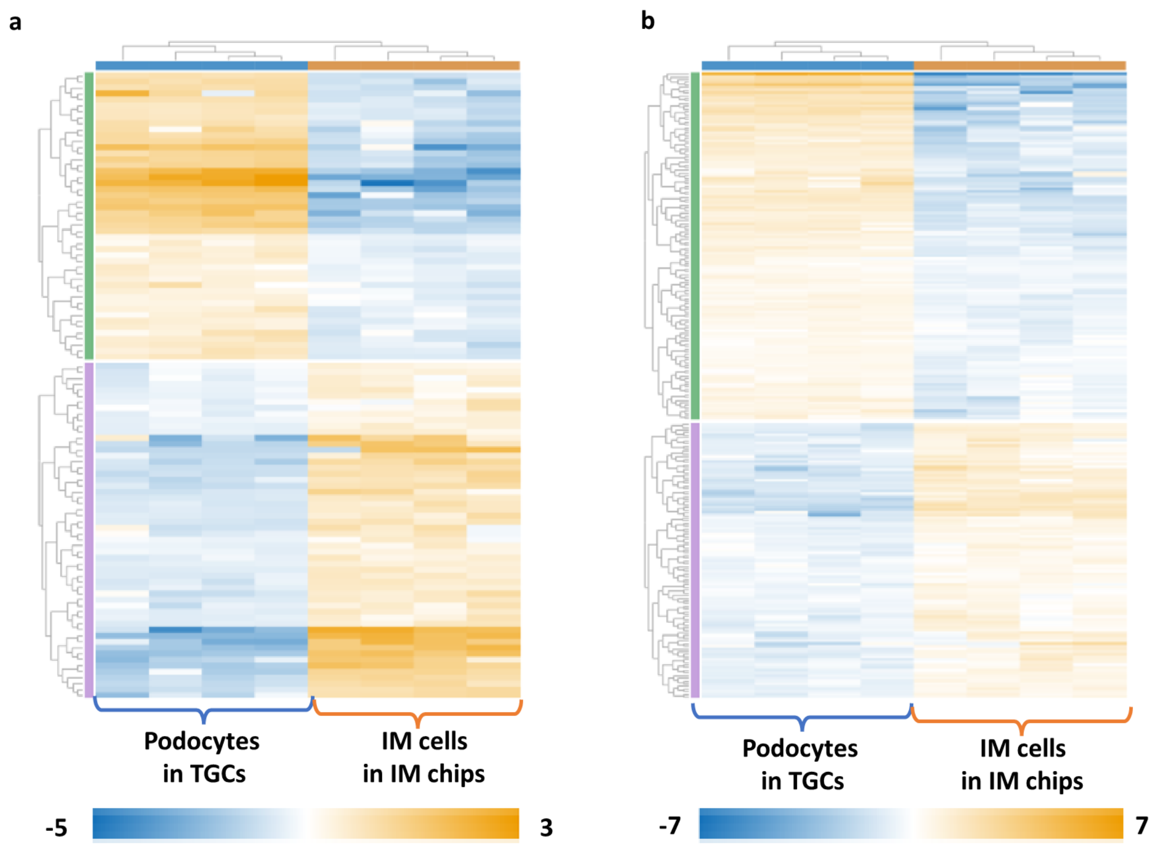

Figure S6. Effect of podocyte differentiation on IM cells. a,b)Two-color heatmaps with color bar show enrichment scores normalized across all samples for the (a) Hallmark of epithelial to mesenchymal transition and (b) TGFβ regulation of ECM pathways in IM cells compared with podocytes in TGCs.

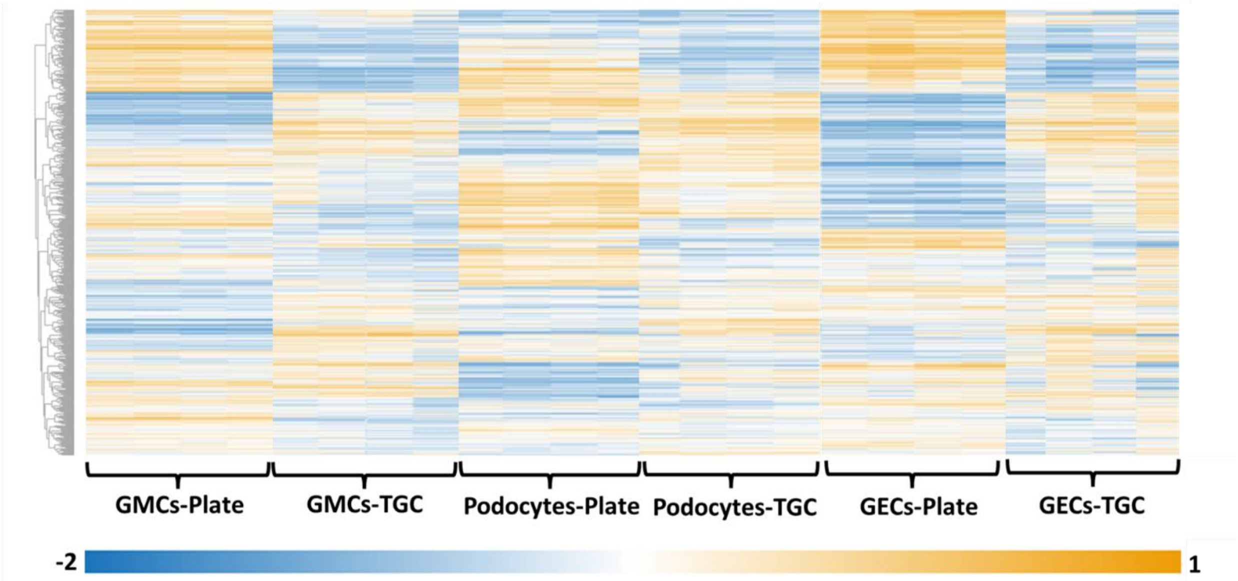

Figure S7. Heat maps of the enrichment scores with color bar. Two-color heatmaps show enrichment scores from Ontology collection/MSigDB Biological Process used for pathway analysis between cells cultured in monoculture and TGC (GECs, glomerular endothelial cells; GMCs, glomerular mesangial cells; TGC, tri-culture glomerular chip).

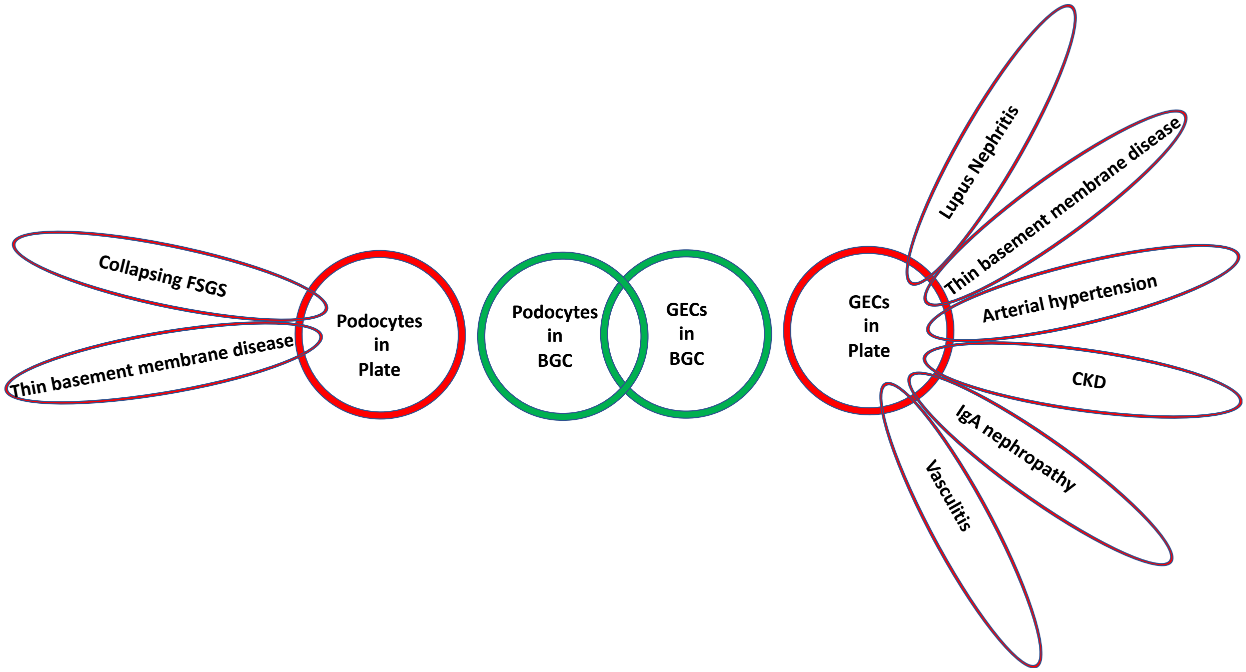

Figure S8. Comparison of disease association of glomerular cell types between co-culture glomerular chip and monocultures. Nephroseq analysis of glomerular cells (GECs and podocytes) cultured in monoculture or in co-culture glomerular chips (BGCs). Disease associations shown in ovals.

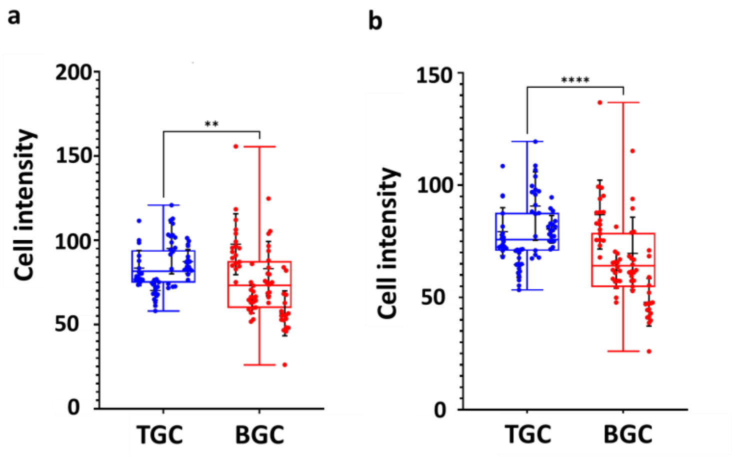

Figure S9. High throughput quantification of nephrin in tri-culture and co-culture chips. a) Nephrin expression in podocytes in chips. b) Membrane-associated nephrin expression in podocytes in chips. n=4 independent chips with 18 selected areas per chip and total of 72 readings for each bar in graphs. Unpaired Mann-Whitney test was used for comparing two columns in each graph; Kruskal-Wallis test and Dunn's multiple comparisons test were used for all comparisons in each graph. $^{**}P < 0.01$ and $^{****}P < 0.001$. Error bars represent mean ± SD.

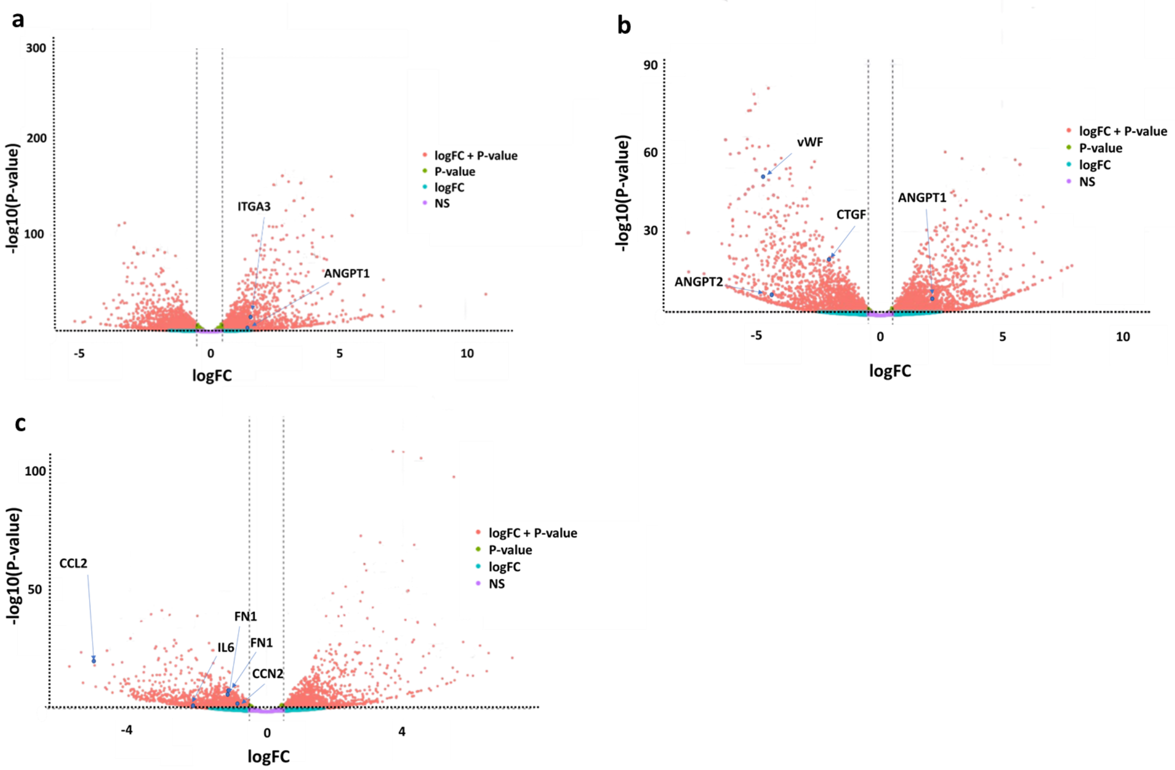

Figure S10. A volcano plot analysis of differential expression analysis. Identifying significantly altered genes between mature TGC and immature TGC (TGC containing immature podocytes (i.e., intermediate mesoderm (IM) cells)). The Y-axis of the volcano plot represents the negative base-10 logarithm (-log10) of the statistical significance (P-values), while the X-axis displays the log2-fold change values of gene expression between these groups. a) altered podocyte's genes. b) altered GEC's genes. c) altered GMC's genes. The genes highlighted in red on the volcano plot are those that exhibit a log2-fold change (Log2FC) greater than 1 or less than -1, representing a twofold or greater difference, and a P value less than 0.05, indicating that the difference in expression between Group A and Group B is statistically significant.

Table S1. Top-ranked disease associated pathways (from gene ontology collection MSigDB Biological Process), and their P values for comparisons between TGC and IM chips and TGC-IM vs BGC-IM

Comparison	Cell type	Pathway	*P* value
TGC vs. IM	Podocytes	Renal system vasculature development	1.80e-04
		Glomerular mesangium development	9.36e-03
		PI3K signaling pathway	7.10e-04
		Response to RA	1.60e-02
		Response to TGF-β pathway	1.04e-02
		Cell division	5.00e-09
		Negative regulation of cell proliferation pathway	4.00e-08
		TGF-β regulation of ECM	≤1.00e-32
		Epithelial mesenchymal transition	5.10e-10
	Endothelial cells	PI3K signaling pathway	8.50e-06
		VEGF signaling pathway	6.90e-07
		VEGF production	1.87e-02
		Cellular response to VEGF stimulus	9.40e-06
		Regulation of cellular response to VEGF stimulus	1.08e-03
		VEGFR signaling pathway	1.14e-03
		Regulation of VEGFR signaling pathway	1.30e-04
		DNA damage response signal transduction by P53 class mediator	6.37e-03
		Response to TGF-β	2.40e-06
		TGF-βR signaling pathway	6.10e-04
		EGFR signaling pathway	1.27e-02
		Positive regulation of NF-kB transcription factor activity	2.18e-02
		Positive regulation of I-kB Kinase NF-kB signaling	1.10e-04
		I-KB kinase NF-KB signaling	6.50e-05
		Response to TNF	7.46e-03
		Positive regulation of IL-8 production	3.44e-02
		Negative regulation of IFN-γ production	5.10e-03
		Response to IFN-γ	7.10e-04
		IFN-γ mediated signaling pathway	1.11e-02
		Response to retinoic acid	4.10e-05
	Mesangial cells	PI3K signaling	9.24e-03
		Regulation of PI3K signaling	4.67e-03
		Positive regulation of NF-kB transcription factor activity	3.47e-03
		Positive regulation of I-kB kinase NF-kB signaling,	2.00e-04
		I-kB kinase NF-kB signaling	7.60e-04
		Regulation of TGF-β activation	2.43e-03
		Regulation of cellular response to TGF-β stimulus	2.90e-02
		Response to TGF-β	1.54e-03
		TGF-β activation	1.07e-02
		TGF-β production	7.26e-03
		Negative regulation of TGF-β production	1.07e-02
		Signal transduction by P53 class mediator	1.62e-02
		DNA damage response signal transduction by P53 class mediator	9.80e-04
		Response to TNF	4.45e-03
		TNF mediated signaling pathway	1.24e-02
		Regulation of TNF mediated signaling pathway	1.44e-02
		Cellular response to VEGF stimulus	3.35e-03
		Regulation of VEGFR signaling pathway	1.66e-03
		Positive regulation of IL-8 production	3.02e-02
		Negative regulation of hypoxia induced intrinsic apoptosis signaling pathway	3.97e-02
		Response to retinoic acid	2.40e-04
TGC-IM vs. BGC-IM	Podocytes	Mitotic cell cycle	5.80e-25
		Cell cycle process	1.40e-24
		Mitotic cell cycle process	2.00e-24
		Cell division	6.00e-16
		Cell cycle	2.50e-22
		DNA replication	2.00e-20

	Lipid metabolic process	1.90e-09
	Cellular lipid metabolic process	4.70e-09
	Electron transport chain (OXPHOS system in mitochondria)	8.80e-04
	Oxidative phosphorylation	3.10e-04
	Oxidation-reduction process	9.30e-12
	Positive regulation notch signaling pathway	1.96e-02
	Cell projection assembly	5.64e-03
	Cell projection organization	2.85e-03
	Cytoskeleton organization	8.20e-06
	Macroautophagy	1.30e-07
Endothelial cells	VEGFA-VEGFR2 signaling	4.50e-06
	TGF-β signaling pathway	8.80e-04
	Endothelial cell proliferation	8.60e-07
	Endothelial cell migration	2.20e-07
	Endothelial cell development	3.37e-03
	Endothelial cell fate commitment	1.32e-02
	Endothelium development	4.80e-07
	Endothelial cell apoptotic process	4.91e-03
	Inflammatory response	≤ 1.00e-32
	Immune effector process	3.60e-05
	Innate immune response	7.80e-05
	TNF-α signaling pathway	2.29e-03
	Cytokine mediated signaling pathway	6.60e-09
	Response to cytokine	1.00e-06
	Macroautophagy	3.20e-07
	Biological adhesion	1.30e-06
	Regulation of cell adhesion	8.80e-04
	Cell-cell adhesion	1.22e-02
	Extracellular structure organization	2.30e-07

Supplementary Videos

Video S1. Acellular 3D fabricated mesangium and its lumen (green, NHS-Ester dye). The FIJI (ImageJ, NIH) software was used to project a sequence of ten immunofluorescent images (stacks) of stained acellular hydrogel.

Video S2. Representative confocal z-stack images of the cellular 3D fabricated mesangium (αSMA in green, VE-Cad in red, and nuclei in blue).

Video S3. Light microscopy view of the bottom channel for better visualization of the location of glomerular mesangial cells (GMC) and glomerular endothelial cells (GEC) in TGCs.
